# Simultaneous Reduction in Noise and Cross-Contamination Artifacts for Dual-Energy X-Ray CT

**DOI:** 10.1155/2013/417278

**Published:** 2013-06-19

**Authors:** Baojun Li, Baohong Li, Jack Luo, Peng Tang, Jiandong Mao, Xiaoye Wu

**Affiliations:** ^1^Department of Radiology, Boston University Medical Center, Boston, MA 02118, USA; ^2^Department of Computer Science & Technology, Xi'an Jiaotong University, Xi'an, Shaanxi 710049, China; ^3^Department of Radiology, Xijing Hospital, Xi'an, Shaanxi 710049, China; ^4^CT System Lab, GE Global Research Center, Schenectady, NY 12309, USA

## Abstract

*Purpose.* Dual-energy CT imaging tends to suffer from much lower signal-to-noise ratio than single-energy CT. In this paper, we propose an improved anticorrelated noise reduction (ACNR) method without causing cross-contamination artifacts. *Methods.* The proposed algorithm diffuses both basis material density images (e.g., water and iodine) at the same time using a novel correlated diffusion algorithm. The algorithm has been compared to the original ACNR algorithm in a contrast-enhanced, IRB-approved patient study. Material density accuracy and noise reduction are quantitatively evaluated by the percent density error and the percent noise reduction. *Results.* Both algorithms have significantly reduced the noises of basis material density images in all cases. The average percent noise reduction is 69.3% and 66.5% with the ACNR algorithm and the proposed algorithm, respectively. However, the ACNR algorithm alters the original material density by an average of 13% (or 2.18 mg/cc) with a maximum of 58.7% (or 8.97 mg/cc) in this study. This is evident in the water density images as massive cross-contaminations are seen in all five clinical cases. On the contrary, the proposed algorithm only changes the mean density by 2.4% (or 0.69 mg/cc) with a maximum of 7.6% (or 1.31 mg/cc). The cross-contamination artifacts are significantly minimized or absent with the proposed algorithm. *Conclusion.* The proposed algorithm can significantly reduce image noise present in basis material density images from dual-energy CT imaging, with minimized cross-contaminations compared to the ACNR algorithm.

## 1. Introduction

Dual-energy X-ray CT permits retrospective decomposition of anatomy into basis material density maps (images) from the low- and high-kVp acquisitions [1–8]. Through material decomposition, the energy-dependent attenuation measurements contained in kVp projections are transformed into energy-independent basis material projection data corresponding to the basis material pair (e.g., water and iodine). Although the pair of basis material projection data (sinogram) essentially contain all useful information about the material being imaged, they are difficult to understand and to be interpreted by physicians. A more useful form, which physicians are familiar with, is the reconstructed images. Having the identical geometry, the same reconstruction algorithm to reconstruct the single-energy CT images can therefore be applied to the first basis material projection data to obtain the corresponding basis material density image. The step is repeated for the second basis material as well. 

**Figure 1:**
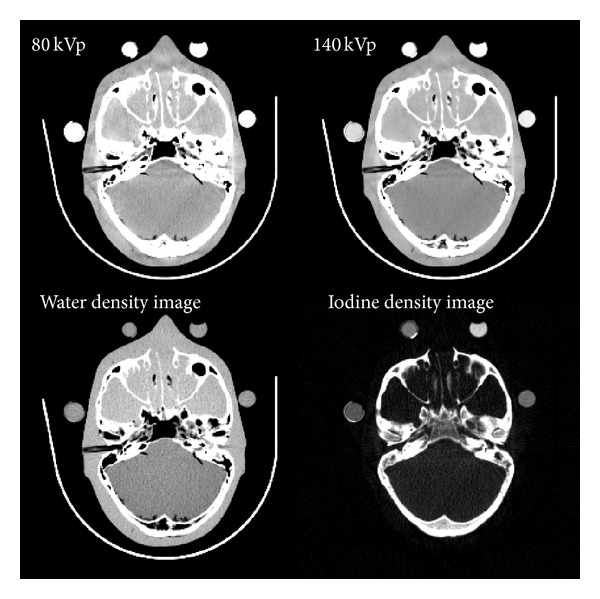
Exemplary reconstructed basis material density images, as well as the kVp images, from a dual-energy head CT exam.

An example is shown in Figure 1. Although the basis material density images may look like the reconstructed kVp images, they represent the effective density (in mg/cc) necessary to create the observed low- and high-kVp attenuation measurements. For instance, pure water appears as 1,000 mg/cc in a water density image, and 50 mg/cc of diluted iodine is labeled as such in an iodine density image, and so forth. In addition, any nonbasis material is mapped to both basis materials. For this reason, basis material density images are sometimes called “material density maps.” 

It is well known that the basis material density images suffer from much lower signal-to-noise ratio (SNR) than single-energy CT images. This can be easily demonstrated by the following simple analysis. Let us define the SNR of iodine in a low-kVp image as

(1) $$\begin{array}{c} \text{SN}{\text{R}}_{L}\left(x,y\right)\propto \frac{\mu_{L}^{I}\left(x,y\right)}{\sigma_{L}\left(x,y\right)}, \end{array}$$

where *μ* ^*I*^ stands for the attenuation coefficient of iodine, *σ* is the noise, (*x*, *y*) represents a pixel coordinate in the image, and subscript *L* indicates the low-kVp image. Then the SNR of iodine in a basis material density image is

(2) $$\begin{array}{c} \text{SN}{\text{R}}_{\Delta E}\left(x,y\right)\propto \frac{\mu_{L}^{I}\left(x,y\right)-w^{I}\mu_{H}^{I}\left(x,y\right)}{\sqrt{\sigma_{L}^{2}\left(x,y\right)+{\left(w^{I}\right)}^{2}\sigma_{H}^{2}\left(x,y\right)}}, \end{array}$$

where *w* ^*I*^ is weighting coefficient to produce the iodine density image, and subscript *H* indicates the high-kVp image. By comparing (2) with (1) and using the fact that *μ*_*L*_^*I*^ and *μ*_*H*_^*I*^ are very close for the most of clinically relevant energy levels, we can conclude that

(3) $$\begin{array}{c} \text{SN}{\text{R}}_{\Delta E}\left(x,y\right)\ll \text{SN}{\text{R}}_{L}\left(x,y\right). \end{array}$$

Noise reduction in dual-energy CT has been an active research area to obtain basis material density images of diagnostic quality. An excellent overview of algorithmic approaches can be found in [9]. Other methods based on the optimization of acquisition protocol and/or hardware have also been proposed [10, 11]. It has been recognized that, however, the most effective noise reduction method for basis material density images exists in anticorrelated noise reduction technique (ACNR) [9, 12–16]. This technique is based on the knowledge that image noises between the basis material density image pair are anticorrelated [1, 12, 17]. Taking advantage of this physical property, Kalender et al. has proposed to use a high-pass filtered version of the first basis material density image (e.g., water) to noise reduce the complimentary basis material density image (e.g., iodine) [12]. In practice, an adaptive filter has replaced the simple high-pass filter in several clinical applications [9, 12–14]. 

**Figure 2:**
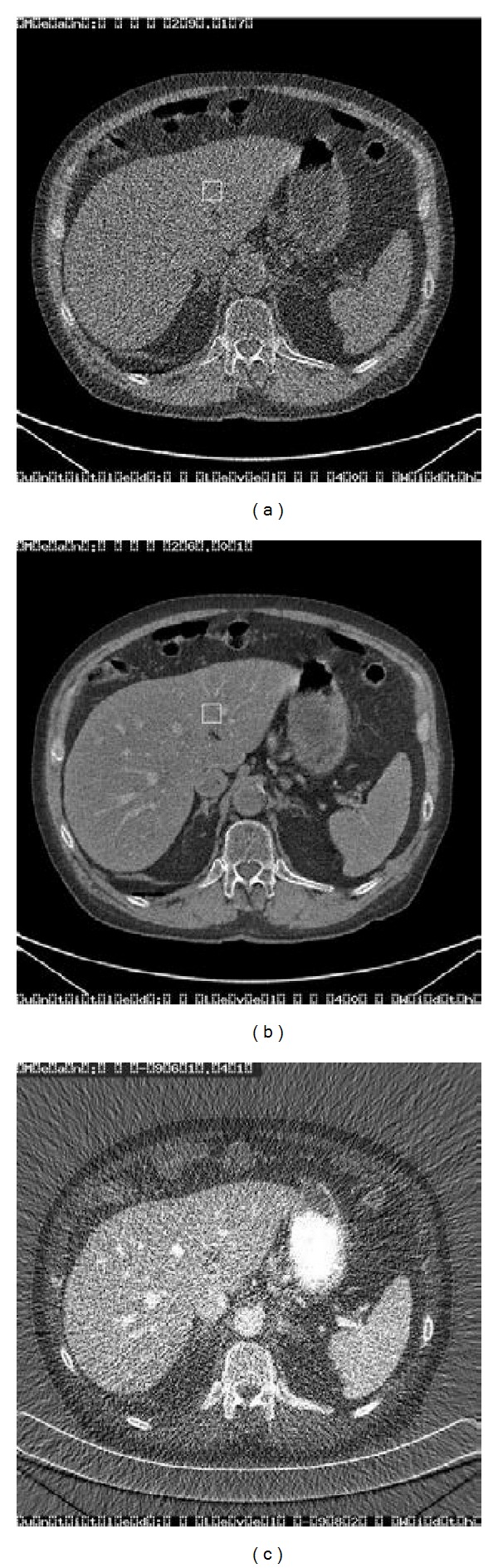
Comparison of the original and noise-reduced basis material (water) density images from a dual-energy abdominal CT exam of a patient. (a) Original water density image. (b) Noise-reduced water density image using the ACNR algorithm [12]. In spite of the fact that the noise in the image is reduced, artifacts are evident throughout the liver area. (c) Original iodine density image. The iodinated hepatic vessels correlate well with the artifacts in the water density image, suggesting that the artifacts are caused by cross-contamination.

ACNR algorithms are effective in suppressing noise. However, they are at the risk to introduce a detrimental artifact [9, 12].  Figure 2 shows a comparison of the original and ACNR noise-reduced water density images from a dual-energy abdominal CT exam of a patient. Although the noise is reduced, artifacts are evident throughout the liver area. By inspecting the iodine density image, it is clear that the iodinated hepatic vessels correlate well with the artifacts in the water density image, suggesting that, during the noise reduction, edge structures and hepatic vessels containing contrast medium were transferred from the iodine density image to the water density image. This artifact will be referred to as “cross-contamination” in this paper. 

Cross-contamination is very undesirable as it alters the original density values and introduces false anatomical or pathological information to the complimentary basis material density image. It not only hinders the quantification accuracy of dual-energy CT imaging, but also potentially leads to misdiagnosis [9]. 

In this paper, we propose an improved ACNR algorithm, based on correlated anisotropic diffusion, which can simultaneously reduce the image noise and minimize the cross-contamination in the basis material density images. Our algorithm can accomplish both tasks at the same time because of the novel design of a correlated anisotropic diffusion filter that intelligently differentiates the correlated anatomical structures from the uncorrelated ones. Although our filter kernel is based on the anisotropic diffusion filters that have gained popularity in medical imaging applications [18–21], the correlated filter kernel design has never been seen in the literatures. Since our algorithm performs diffusion in both basis material density images simultaneously, it is also more efficient than the original ACNR algorithms. 

## 2. Methods

### 2.1. Anisotropic Diffusion Filter

The traditional gradient-based denoising model could not retain the image details well. Anisotropic diffusion-based filters represent the most promising denoising technique besides statistical iterative reconstruction, but do not rely on proprietary CT projection data that is often not retrievable retrospectively [18]. To fully understand our proposed algorithm, we need to briefly describe the anisotropic diffusion filter first. 

The diffusion operation can be described by the following equation:

(4) $$\begin{array}{c} \frac{\partial I\left(x,y,t\right)}{\partial t}=\mathrm{div}\left[D\left(\nabla I\right)\right], \end{array}$$

where ∇*I* denotes the local image gradient and the operator *D*  the diffusive function. Present anisotropic diffusion filter constructs *D* by either a simple signal-to-noise ratio (SNR) measure or, more sophistically, a measure of the local contour and gradient principal directions and their relative strength [19]. 

The diffusive function should be monotonically decreasing so that diffusion decreases as the gradient increases. One of such function is

(5) $$\begin{array}{c} D\left(\nabla I\right)=\alpha \cdot e^{-{||\nabla I||}^{2}/2\sigma^{2}}. \end{array}$$

Equations (4) and (5) describe an iterative process in which the diffusion operation continues until a stop criterion is reached. The parameter *σ* is estimated from the noise in the image. Owing to its edge preserving power, anisotropic diffusion filters have been widely used in many medical imaging applications as a noise and speckle reduction tool [19–21]. 

### 2.2. Correlated Anisotropic Diffusion Filter

In this section, we propose a correlated anisotropic diffusion that simultaneously diffuses both basis material density images:

(6) ∂Im1(x,y,t)∂t=div⁡[D1(∇Im1,∇Im2)],∂Im2(x,y,t)∂t=div⁡[D2(∇Im1,∇Im2)],

where *m*1 and *m*2 denote the two basis materials, *I*_*m*1_ and *I*_*m*2_ the corresponding density images, and *D*_1_ and *D*_2_ the diffusive functions that have the form of

(7) D1(∇Im1,∇Im2)=α·e−f(∇Im1,∇Im2)/2σm12,D2(∇Im1,∇Im2)=β·e−f(∇Im1,∇Im2)/2σm22,

where

(8) f(∇Im1,∇Im2)  =γ·||∇Im1||2+δ·||∇Im2||2   +φ·||∇Im1∇Im2||2+ω·||∇Im2∇Im1||2.

**Figure 3:**
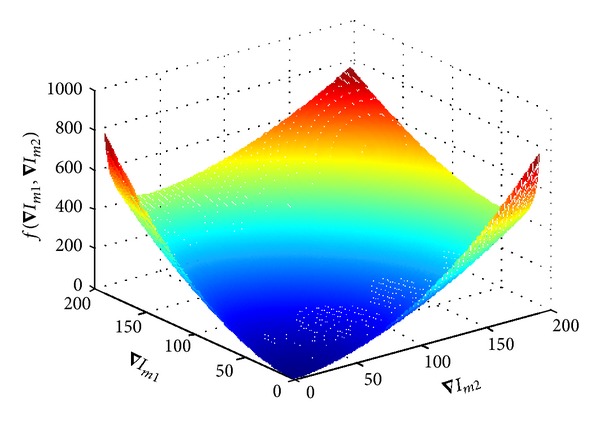
A graphic representation of *f*(∇*I*_*m*1_, ∇*I*_*m*2_) from (8).

A graphic representation of *f*(∇*I*_*m*1_, ∇*I*_*m*2_) is displayed in Figure 3. It is clear from the figure that the diffusion strength decreases when the gradient in either image increases, or the difference in gradient between the two images increases. Since most high spatial resolution features are present in the lung and bony regions, the first two terms in (8) make that sure the filtration in these regions needs to be kept to a minimum to reduce its impact on spatial resolution. On the other hand, since iodinated hepatic vessels appear only in one of the images, the third and fourth terms in (8) ensure that the diffusion strength is significantly reduced as well around these vessels to avoid cross-contamination. 

Through (7), correlated diffusion is performed on both images simultaneously. To improve computational speed, we assess (7) in a multiresolution fashion. In another word, we first downsample the CT images to 128 × 128 pixels and perform correlated diffusion on the down-sampled images. The resulting images are then upsampled to 256 × 256 pixels and 512 × 512 pixels, with correlated diffusion performed on each resolution. To preserve the image resolution, a lossless wavelet similar to that used in [20] has been employed to perform the down- and upsamplings. A prototype software has been developed for this study in PV-WAVE Rogue Wave Software, (Boulder, CO) on a standard Red hat Linux system with Intel dual-core CPU (3.3 GHz). The computation speed is roughly 0.7 second per slice (512 × 512 pixels). 

### 2.3. Noise Suppression

Next, we compute a noise mask for each basis material density image that is simply the difference between the original image, *I*_*m*1_  (or *I*_*m*2_), and its filtered version (i.e., the resultant image from the correlated anisotropic diffusion step described in Section 2.2) as follows:

(9) Nm1(x,y)=Im1(x,y)−∂Im1(x,y,t)∂t,Nm2(x,y)=Im2(x,y)−∂Im2(x,y,t)∂t.

Finally, we follow the general scheme of ACNR algorithm to cancel the noise in a basis material density image using the weighted complimentary noise mask [12] as follows:

(10) Im1′(x,y)=Im1(x,y)+μm2(E0)μm1(E0)·Nm2(x,y),Im2′(x,y)=Im2(x,y)+μm1(E0)μm2(E0)·Nm1(x,y),

where *μ*_*m*1_ and *μ*_*m*2_ denote the linear attenuation coefficients of materials  *m*1 and *m*2, respectively, and *E*_0_ symbolizes the optimal energy at which the anticorrelated noises are best cancelled, resulting in the highest SNR in the monochromatic energy images [8]. 

### 2.4. Experiment

The proposed algorithm has been applied to dual-energy abdominal CT exams of five patients to evaluate its efficacy. The patient population includes three males and two females, with the age ranging from 21 to 63 years old. The average patient weight is 57.6 kg. The human study has been approved by the Institutional Review Board (IRB) of Xijing Hospital, Xi'an, China. 

The patients are scanned on a GE 750HD CT scanner (GE Healthcare, Waukesha, WI) that features the fast-kVp switching dual-energy capability [8]. The dual-energy protocol includes alternating the tube potential between 80 and 140 kVp on a view-by-view basis, 600 mA, and 0.6 sec gantry rotation time, large body bowtie, and 40 mm collimation. All datasets are reconstructed with 5 mm slice thickness, standard reconstruction kernel, and a display field of view of 36 cm. The CTDI_VOL_ is 17.64 mGy, which is comparable to that of a single-energy abdominal CT exam. 

The CT scan begins approximately 75 seconds after the patient receives 100 mL Optiray 350 (Covidien Pharmaceuticals, Hazelwood, MO), which is administered intravenously at a fixed rate of 3-4 mL per second. 

## 3. Results

**Figure 4:**
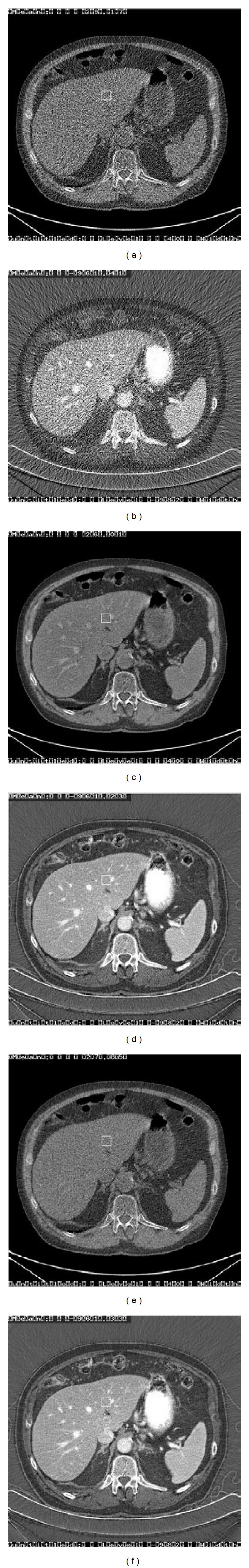
Example images from a dual-energy abdominal CT study. ((a) and (b))  Original water and iodine density images of suboptimal image quality due to its high noise level. ((c) and (d)) Noise-reduced water and iodine density images using the ACNR algorithm [12]. Cross-contamination artifacts are clearly visible throughout the liver area in (c). The iodinated hepatic vessels in (d) are the root cause of the cross-contaminations seen in (c). ((e) and (f)) Noise-reduced water density images using the proposed algorithm which are free of contamination.

Figures 4(a) and 4(b) depict the original water and iodine density images from the same abdominal exam showed earlier in Figure 2. The image quality is suboptimal due to its high noise level. The corresponding noise-reduced density images using the proposed algorithm are shown in Figures 4(e) and 4(f). It is clear that the proposed algorithm has significantly reducing the noise and thus enhanced the image quality. For comparison, the noise-reduced density images using the ACNR algorithm are also displayed in Figures 4(c) and 4(d). By comparing Figures 4(c) and 4(e), it is obvious that the proposed algorithm is superior to the ACNR algorithm in minimizing the contamination artifacts. 

**Figure 5:**
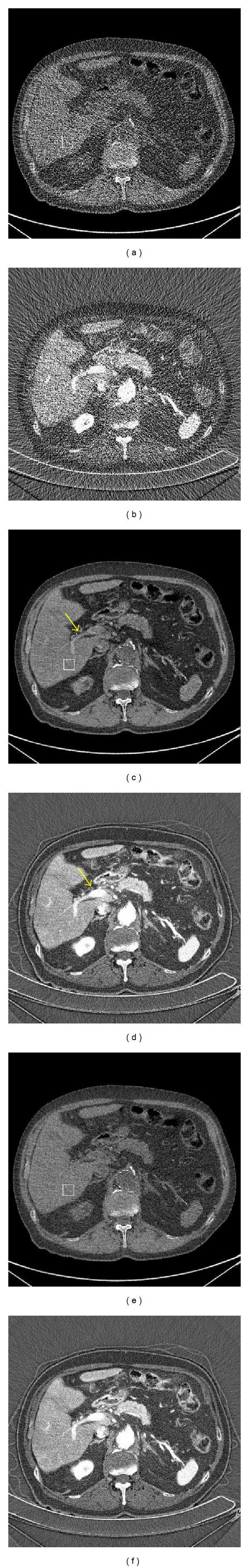
Example images from a dual-energy liver study. ((a) and (b)) Original water and iodine density image. ((c) and (d)) Noise-reduced water and iodine density images using the ACNR algorithm [12]. Cross-contamination artifacts are clearly visible near the main hepatic portal vein (arrow) in (c). The iodinated hepatic portal vein (arrow) in (d) is the root cause of the cross-contamination seen in (c). ((e) and (f)) Noise-reduced water density images using the proposed algorithm which are free of contamination.

Figures 5(a) and 5(b) depict the noisy original water and iodine density images from another abdominal study. The corresponding noise-reduced density images using the ACNR algorithm are displayed in Figures 5(c) and 5(d). Cross-contamination artifacts can be easily seen near the main hepatic portal vein (pointed by arrow) in the water density image. By inspecting the complimentary iodine density image, the iodinated hepatic portal vein (pointed by arrow) in Figure 5(d) is the root cause of the cross-contamination. Figures 5(e) and 5(f) show the noise-reduced density images using the proposed algorithm. It is clear that the proposed algorithm can minimize the cross-contamination artifacts while significantly reduced the noise in both density images. 

Table 1 summarizes the quantitative results from the clinical cases (including the two cases shown in Figures 4 and 5). We define the material density accuracy and the amount of noise reduction by a percent density error,

(11) $$\begin{array}{c} \%\;\text{density}\;\text{error}=\frac{\text{D}{\text{ensity}}_{\text{original}}-{\text{Density}}_{\text{noise}\;\text{reduced}}\;}{{\text{Density}}_{\text{original}}}, \end{array}$$

and a percent noise reduction,

(12) $$\begin{array}{c} \%\;\text{noise}\;\text{reduction}=\frac{\;\delta_{\text{original}}-\delta_{\text{noise}\;\text{reduced}}\;}{\delta_{\text{original}}}, \end{array}$$

where *δ* symbolizes the standard deviation. Both material density and standard deviation are measured in a ROI (20 × 20 pixels) in the relative smooth liver region, such as those shown in Figures 4 and 5. From Table 1, it can be seen that the average percent noise reduction is 69.3% and 66.5% with the ACNR algorithm and the proposed algorithm, respectively. In addition, the material densities are changed by an average of 13.1% and 2.4% after the ACNR algorithm and the proposed algorithm, respectively, compared to the original densities. 

**Figure 6:**
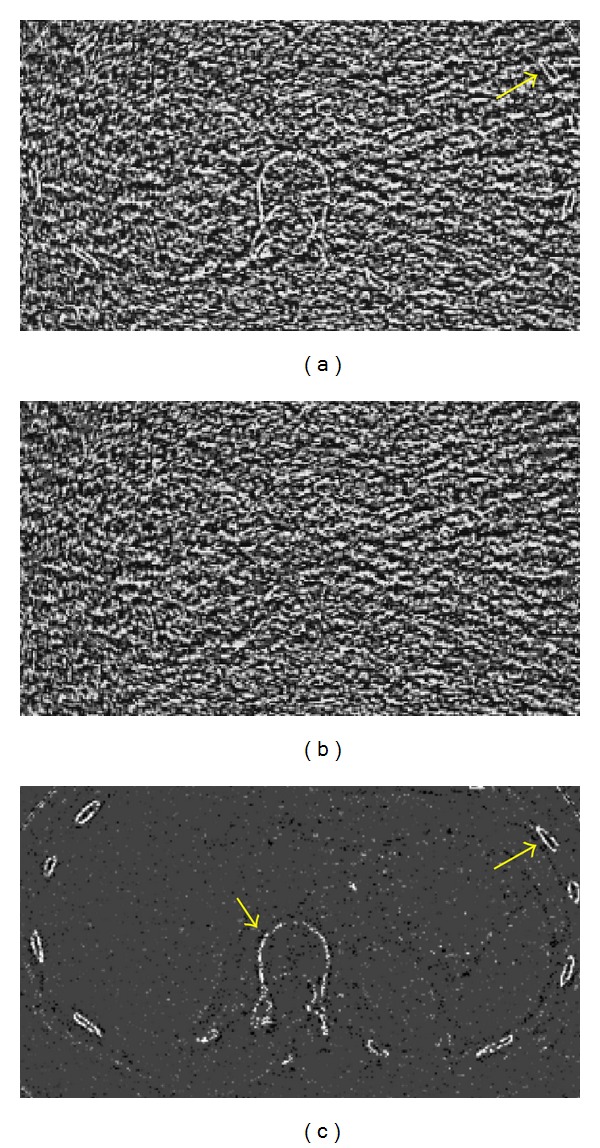
Example of noise masks randomly selected from one of the clinical exams. (a) Noise mask generated using the ACNR algorithm [12]. Structures such as vertebrae and ribs can be clearly seen (arrows). (b) Noise mask created using the proposed algorithm. (c) The difference image between (a) and (b). Reduction of anatomical structures is evident with the proposed algorithm.

Figure 6 compares noise masks randomly selected from one of the patient exams. As indicated in (9), a noise mask is computed as the difference between the original water (or iodine) density image and its filtered version. Figures 6(a)–6(c) display the noise mask generated using the ACNR algorithm, the noise mask generated using the proposed algorithm, and the difference image, respectively.  Figure 6(a) contains a large amount of edge structures, such as ribs and vertebrae (arrows), suggesting a high likelihood of cross-contamination when the noise mask is used to denoise the complimentary basis material density image. On the contrary, these anatomical structures are absent from Figure 6(b). The reduction of contamination-prune structures is evident as shown in the difference image (Figure 6(c)). This further exemplifies the proposed algorithm's ability to minimize cross-contaminations.

## 4. Discussions

Table 1 shows that both algorithms—ACNR and our proposed—can significantly reduce the noises of basis material density images derived from material decomposition. As a result, the image quality, and potentially diagnosis efficacy, is substantially improved (e.g., Figure 4(e) versus Figure 4(a)). Since a noise reduction by a factor of *n* would otherwise require an exposure increase by *n* ^2^, either algorithm may lead to tremendous dose saving to patients who undergo dual-energy CT exams. 

However, the noise reduction by the ACNR algorithm is at a cost of altering the material density by, for example, an average of 13% (or 2.18 mg/cc) with a maximum of 58.7% (or 8.97 mg/cc) in this study. These alterations are clinically and quantitatively undesirable. Although it is difficult to avoid introducing cross-contaminations, we challenge ourselves to minimize this adversity while preserving the ACNR algorithm's effectiveness in noise suppression. 

As shown in Table 1, the proposed algorithm only changes the mean density by 2.4% (or 0.69 mg/cc) with a maximum at 7.6% (or 1.31 mg/cc). This is also evident from the cases shown in Figures 4 and 5. In both cases, ACNR algorithm has resulted in massive cross-contamination artifacts, particularly in the water density images. The artifacts, however, are minimized or absent after the proposed algorithm. It should be noted that this minimization of cross-contamination comes at a small cost—the average percent noise reduction decreases from 69.3% to 66.5%. However, compared to retaining the material density accuracy, we feel that this slightly increased noise is acceptable. 

Although anisotropic diffusion has gained its popularity since the 1990s [18–21], the correlated anisotropic diffusion algorithm has never been described in the literature. In this work, we take advantage of the well-known noise property of dual-energy CT imaging (anticorrelation) [1, 12, 17] and incorporate it into our anisotropic diffusion framework. The unique formulation is shown to have improved the overall quantitative accuracy of the basis material densities compared to high-pass filter-based approaches [12–14]. Other noise suppression approaches, such as noise forcing and clipping, have also attempted to correlate the water and iodine (or water and bone) densities [15]. The proposed algorithm differs from these approaches in that our approach is based on neighborhood operation (i.e., diffusion) and thus does not leave any pixilated noise appearance near edge pixels or in area with high noise levels. 

The dual-energy protocol used in this study results in 47% lower dose compared to the manufacturer's default protocol (i.e., 17.6 mGy versus 33.4 mGy [8]). Our protocol is selected to satisfy an IRB requirement that the CTDI_VOL_ from dual-energy exams should be consistent with that of single-energy abdominal CT exams. The consequence is that the original basis material density images are fairly noisy, as evidenced from Figures 4(a) and 4(b) and Figures 5(a) and 5(b). Nevertheless, the focus of this study is not the optimization of scanning protocol; thus this is still considered acceptable. For the same reason, we did not correct CTDI_VOL_ for individual patient sizes [22]. 

One limitation of this study is that there is no observer study-based image quality assessment. As a result, the benefit of overall image quality enhancement from either the ACNR algorithm or the proposed algorithm cannot be quantified compared to the original material density images. This is one of the future directions we will be working on. 

## Figures and Tables

**Table 1:** Percent CT number accuracy and percent noise reduction observed in five clinical cases. Both CT number and standard deviation are measured in a ROI (20 × 20 pixels) in the relative smooth liver region, such as those shown in Figures 4 and 5. ACNR: anticorrelated noise reduction [12].

Dataset	Basis material	Algorithm	Mean density (mg/cc)	Standard deviation	Absolute density error (mg/cc)	% Density error	% Noise reduction
Case 1 (Figure 4)	Water	Original	29.16	72.22	—	—	—
		ACNR	26.00	24.26	3.16	10.8%	66.4%
		Proposed	27.85	30.29	1.31	4.5%	58.1%
	Iodine	Original	−961.41	29.71	—	—	—
		ACNR	−961.22	9.45	0.19	0.002%	68.2%
		Proposed	−961.33	10.32	0.08	0.008%	65.3%

Case 2 (Figure 5)	Water	Original	15.28	88.27	—	—	—
		ACNR	24.25	27.99	8.97	58.7%	68.3%
		Proposed	15.42	28.17	0.14	0.9%	68.0%
	Iodine	Original	−967.92	31.42	—	—	—
		ACNR	−967.41	9.83	0.51	0.05%	68.7%
		Proposed	−966.31	10.98	0.61	0.2%	65.1%

Case 3	Water	Original	5.74	85.39	—	—	—
		ACNR	6.81	25.99	1.07	18.6%	69.6%
		Proposed	5.93	27.07	0.19	3.7%	68.3%
	Iodine	Original	−934.50	34.77	—	—	—
		ACNR	−935.87	9.26	1.37	0.2%	73.2%
		Proposed	−935.61	9.91	1.11	0.2%	71.5%

Case 4	Water	Original	9.42	45.94	—	—	—
		ACNR	7.48	15.89	1.94	20.6%	65.4%
		Proposed	8.70	16.57	0.72	7.6%	63.9%
	Iodine	Original	−993.72	12.84	—	—	—
		ACNR	−994.56	3.41	0.84	0.08%	73.4%
		Proposed	−994.25	3.94	0.53	0.05%	69.3%

Case 5	Water	Original	12.98	161.68	—	—	—
		ACNR	15.81	48.47	2.83	21.8%	70.0%
		Proposed	13.85	51.20	0.87	6.7%	68.3%
	Iodine	Original	−990.19	20.36	—	—	—
		ACNR	−989.32	6.25	0.87	0.09%	69.3%
		Proposed	−988.89	6.76	1.3	0.1%	66.8%

		Original	—	—	—	—	—
Average		ACNR	—	—	**2.18**	**13.1%**	**69.3%**
		Proposed	—	—	**0.69**	**2.40%**	**66.5%**

		Original	—	—	—	—	—
Maximum		ACNR	—	—	**8.97**	**58.7%**	**73.4%**
		Proposed	—	—	**1.31**	**7.6%**	**71.5%**
